# Biomarkers for Gastroesophageal Reflux in Respiratory Diseases

**DOI:** 10.1155/2013/148086

**Published:** 2013-04-09

**Authors:** Össur Ingi Emilsson, Þórarinn Gíslason, Anna-Carin Olin, Christer Janson, Ísleifur Ólafsson

**Affiliations:** ^1^Faculty of Medicine, University of Iceland, Vatnsmyarvegur 16, 101 Reykjavik, Iceland; ^2^Department of Respiratory Medicine and Sleep, Landspitali University Hospital, 108 Reykjavik, Iceland; ^3^Occupational and Environmental Medicine, Sahlgrenska Academy, Gothenburg University, 413 90 Göteborg, Sweden; ^4^Department of Medical Sciences: Respiratory Medicine and Allergology, Uppsala University, 753 12 Uppsala, Sweden; ^5^Department of Clinical Biochemistry, Landspitali University Hospital, 108 Reykjavik, Iceland

## Abstract

Gastroesophageal reflux (GER) is commonly associated with respiratory symptoms, either through a vagal bronchoconstrictive reflex or through microaspiration of gastric contents. No diagnostic test is available, however, to diagnose when respiratory illnesses are caused by GER and when not, but research in this field has been moving forward. Various biomarkers in different types of biosamples have been studied in this context. The aim of this review is to summarize the present knowledge in this field. GER patients with respiratory diseases seem to have a different biochemical profile from similar patients without GER. Inflammatory biomarkers differ in asthmatics based on GER status, tachykinins are elevated in patients with GER-related cough, and bile acids are elevated in lung transplant patients with GER. However, studies on these biomarkers are often limited by their small size, methods of analysis, and case selections. The two pathogenesis mechanisms are associated with different respiratory illnesses and biochemical profiles. A reliable test to identify GER-induced respiratory disorders needs to be developed. Bronchoalveolar lavage is too invasive to be of use in most patients. Exhaled breath condensate samples need further evaluation and standardization. The newly developed particles in exhaled air measurements remain to be studied further.

## 1. Introduction

Gastroesophageal reflux (GER) is a growing health problem in the Western world [1]. It is now generally accepted that GER is a causative factor for inducing or worsening certain respiratory symptoms and diseases [2]. GER has also been shown to be associated with obstructive sleep apnea [3]. The respiratory diseases that have most frequently been studied with GER are asthma [4–15] and chronic cough [5, 13, 14, 16–23], but recently many studies have been published on GER and lung transplant (LTx) rejection [24–32].

Despite these evident associations, it is difficult to diagnose with certainty when respiratory diseases are caused by GER, or when they cooccur coincidentally. This increases the need for diagnostic methods to discriminate between patients with coincidental cooccurrences and actual causation associations [33]. 

**Figure 1:**
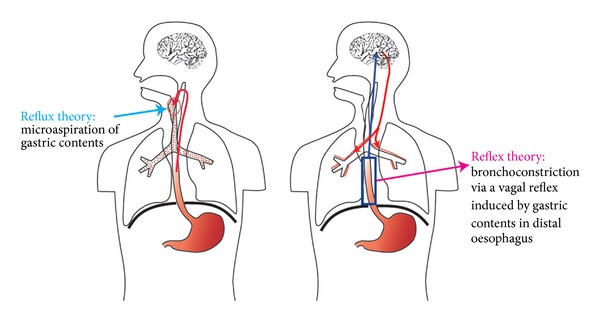
Two theories exist on how gastroesophageal reflux induces respiratory symptoms, called the reflux and reflex theories.

Two different mechanisms are proposed to be responsible for the majority of GER-induced respiratory symptoms and diseases. One involves microaspiration of gastric fluids into the lungs causing irritation and inflammation, and the second bronchoconstriction caused by a vagal reflex from the distal esophagus, induced by acidic reflux to the distal esophagus (Figure 1) [34]. These two mechanisms probably both play a significant role, but to a different extent in different conditions.

Serum biomarkers such as gastrin, pepsinogen, and cleaved fragments of E-cadherin have been studied in patients suffering from GER. Cleaved fragments of E-cadherin were found to be significantly increased in serum from GER patients. As E-cadherin is a junctional protein important in barrier function in esophageal epithelium, its cleavage likely explains the increase in junctional permeability in the esophageal epithelium of these patients [35, 36]. These studies, however, had no focus on respiratory symptoms.

**Figure 2:**
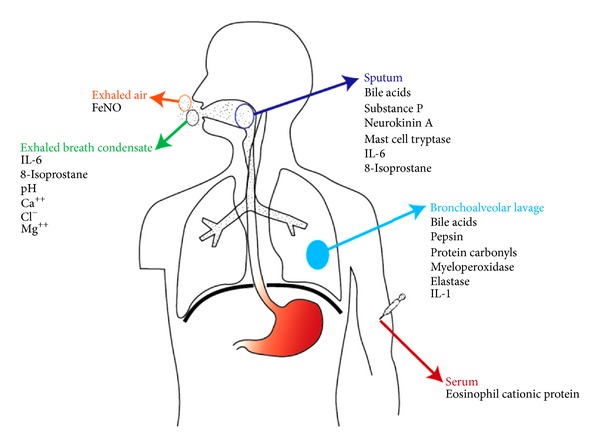
Summary of biomarkers shown to have an association with gastroesophageal reflux in respiratory illnesses.

Clinically it would be of great advantage to have a diagnostic test that could discriminate between respiratory symptoms and diseases caused by GER or other causes. To date, no such generally approved diagnostic test specific to this condition is available, but research in this field has been moving forward (Figure 2) [33]. The aim of the present paper is to summarize the findings of studies on various biomarkers in different biosamples, which have had the objective of distinguishing between respiratory diseases caused by GER- and other non-GER-related causes.

## 2. Methods

When preparing this paper we searched the MEDLINE database for relevant articles on biomarkers associated with GER and respiratory diseases, with special emphasis on biomarkers and biosamples from the distal airways, that is, bronchoalveolar lavage (BAL) and exhaled breath condensate (EBC). The MEDLINE database was searched in August–November 2012 for articles in English. The following search phrases were used: “serum + biomarker + gastroesophageal + reflux” (22 articles), “sputum + respiratory + gastroesophageal + reflux” (34 articles), “bronchoalveolar + lavage + gastroesophageal + reflux” (71 articles), “exhaled + breath + condensate + gastroesophageal + reflux” (16 articles), “exhaled + nitric + oxide + gastroesophageal + reflux” (7 articles), and “particles + in + exhaled + air + gastroesophageal + reflux” (0 articles). Articles were excluded if they contained no abstract or were not relevant (i.e., not studying biomarkers in GER with respiratory symptoms). Review articles, case reports, and letters were also excluded. Animal studies were excluded. Cytopathological studies were mostly excluded, except for the exceptional case of the lipid-laden macrophage index (LLMI), usually in bronchoalveolar lavage, a marker commonly associated with pulmonary aspiration [37–39]. Studies on lung transplant (LTx) patients were included as GER has a special importance in these patients, causing inflammation and transplant rejections [40]. First, titles and abstracts were quickly evaluated with regard to the exclusion criteria, then a closer evaluation of the remaining articles was done. One study was moved from the EBC group to the BAL group [41]. After exclusions, the number of articles identified in each search was as follows: 1, 9, 21, 7, 4, and 0, respectively. No duplicate hits were found. A few studies used more than one biosample and were therefore included in more than one section in this paper (Table 1). 

## 3. Serum Biomarkers

A specific and sensitive serum biomarker for the detection of respiratory disorders caused by or linked to GER has not been found at present. 

In a study by Di Lorenzo et al. [6], patients with GER and asthma-like symptoms were significantly lower in serum eosinophil cationic protein (ECP) levels than patients with diagnosed mild asthma. In fact, those with GER and asthma-like symptoms had similar levels of ECP as healthy subjects, while asthmatic patients had three times higher values. Bronchial hyperresponsiveness was elevated in asthmatic patients, but was normal in those with GER and asthma-like symptoms. The authors hypothesize that this might reflect that those with asthma have mainly eosinophilic inflammation, whereas those with GER have mainly neutrophilic inflammation in the airways. However, this study was cross-sectional, and no follow-up studies have been published [6].

In a study by Chaudhuri et al. [17], serum levels of the neurotrophins nerve growth factor (NGF), brain-derived neurotrophic factor, and neurotrophin 3 were measured in 81 patients suffering from chronic cough and the levels compared to those in healthy controls. No significant association was found between GER-based chronic cough, defined by clinical presentation and treatment response, and these neurotrophins in serum [17].

## 4. Biomarkers in Sputum

Sputum samples contain many biomarkers of inflammation and infection that are relevant for lung diseases and have been proposed to be useful for the detection of respiratory disorders caused by or linked to GER. These markers include bile acids, pepsin, markers for neurogenic inflammation, and general inflammation. 

In a cross-sectional study, bile acids were shown to be frequently present in induced sputum samples in patients suffering from cystic fibrosis. The levels of bile acids were also associated with the degree of lung function impairment [42]. In another study bile acid levels were shown to be significantly elevated in induced sputum from patients with GER and asthma-associated GER symptoms when compared to asthma patients and healthy controls. Patients with asthma had a moderate but statistically insignificant elevation of bile acids in induced sputum samples, both those with and without GER [4]. No statistical difference was observed when bile acid levels in induced sputum from patients with GERrelated chronic cough were compared with sputum samples from controls [18]. In vitro bile acids have been shown to induce fibroblast proliferation in airway epithelium, a finding of unknown importance in humans [4]. There is thus some evidence that bile acids in sputum indicate GER-induced respiratory disorder. However, the pathogenesis behind the association between cough and GER seems to be something other than aspiration of gastric fluids.

Two studies showed that pepsin concentration in sputum is not helpful in diagnosing GER-related chronic cough, and pepsin concentration is frequently detected in sputum from healthy children [18, 19, 43]. One reason why pepsin concentration in sputum might not be as useful as a marker of microaspiration, as originally thought, is that pepsinogen has been found to be produced in normal human lungs [44, 45]. Lipid laden macrophage index (LLMI), a semiquantitative evaluation of macrophage lipid content which is considered to be a biomarker of aspiration, has been studied in the sputum of GER patients with respiratory symptoms. In a small study of 22 patients and 15 controls, the LLMI in sputum was associated with the duration of GER symptoms, but the levels were not significantly different compared with controls [46]. 

In one cross-sectional study, the neurotrophin nerve growth factor (NGF) levels were measured in sputum from patients with chronic cough and compared with healthy controls, but no significant difference was observed. The same was true for the subgroup with chronic cough based on GER [17].

In another study patients with GER and chronic cough were shown to have 50–100 times more of the tachykinin substance P in their sputum when compared to GER patients without chronic cough or healthy subjects [16]. Similarly, a cross-sectional study of 32 subjects showed a positive association between GER and the tachykinins substance P and neurokinin A in induced sputum samples, both in asthmatics and nonasthmatics. A positive correlation between distal esophageal acid exposure time on 24-hour esophageal pH monitoring (24 h-pH-m) and tachykinin levels was found. The presence of these tachykinins suggests airway sensory nerve activation [5]. These findings support the theory that certain respiratory symptoms and diseases linked to GER are caused by a vagally mediated esophageal-tracheobronchial reflex.

The inflammatory biomarker mast cell tryptase has been found to be increased in the sputum of GER-associated chronic cough patients, compared to GER patients without chronic cough. However, other biomarkers of inflammation such as prostaglandin D2 and histamine were not significantly increased in these samples [16]. Another study on inflammatory markers in 20 GER patients with cough or mild asthma found no elevation in fibrinogen levels in induced sputum, and an elevation in ECP was more associated with asthma than with GER. The study was, however, limited by its size [13].

A study on interleukins (IL) and 8-isoprostane in the sputum of GER and asthma patients showed that IL-4 is similarly elevated in asthmatics, with or without GER. Conversely, IL-6 and 8-isoprostane were elevated in GER patients, irrespective of asthma status. Similar findings were found in BAL samples [7].

In summary, bile acid levels in sputum might be associated with GER-induced respiratory disorders. There seems to be a difference in the inflammatory pathways between asthmatics with or without GER. IL-6 and 8-isoprostane, as well as substance P and neurokinin A, in induced sputum seem to predict the presence of GER in subjects both with or without asthma. Substance P and mast cell tryptase seem also to predict GER in subjects with chronic cough. Further studies are needed to clarify these findings.

## 5. Bronchoalveolar Lavage Biomarkers

A bronchoalveolar lavage (BAL) sample is a biosample collected during a bronchoscopy by infusing saline into a small segment of the lung and then suctioning up this fluid again for analysis. The infusion-suction process is then repeated a few times until an adequate specimen has been obtained [47].

Measurements of bile acids in BAL samples consistently show that elevated levels of bile acids are a bad prognostic factor for rejection rates in LTx patients, development of bronchiolitis obliterans, and survival [26, 32]. There seems even to be a clear correlation between the time to onset of bronchiolitis obliterans and levels of bile acids in BAL. In a study by Blondeau et al., nocturnal GER was found to be a stronger risk factor for bile acid aspiration in LTx patients than GER in general, suggesting that nocturnal GER represents a worse form of GER [26, 28–32].

A study on 96 children with chronic cough, allergic asthma, and other chronic lung diseases showed no association between GER status, measured by 24 h-pH-m, and bile acids in BAL [48]. Also, a small study on Wegener's granulomatosis patients with subglottic stenosis showed no significant difference in BAL bile acids; however, since the study examined a very small number of patients, it may be a false negative finding [49].

The clinical use of pepsin as a biomarker in BAL samples has been studied extensively. Many of these studies were on LTx patients. Observations in these studies, however, were somewhat different from those on bile acids in BAL samples. In three of these studies, pepsin in BAL seemed not to be associated with a clinical decline in LTx patients. One recent study, however, showed that pepsin was present in lower quantities in LTx patients who underwent antireflux surgery than in those who did not and was undetectable in controls. Those who underwent antireflux surgery also had a better clinical outcome. This study did not measure bile acids [25, 28, 29, 31]. Another study on 8 LTx patients undergoing antireflux surgery showed a similar trend [24].

Two studies on pepsin in BAL samples from chronic cough patients showed conflicting results. The larger and more thorough one showed no increase in BAL pepsin concentration among chronic cough patients compared with controls, even though they more often had GER, suggesting that aspiration is perhaps not the causative mechanism in GER-associated chronic cough [19, 21]. One study on children with chronic lung diseases showed that those with GER have a higher pepsin level in BAL than those without GER, but with low specificity [48].

Several studies on the clinical use of LLMI in BAL samples have been carried out. Three of them were on children with difficult-to-treat respiratory symptoms, often asthma-like, and one on infants with chronic respiratory diseases. These studies showed a clear association between GER status and LLMI [8, 50–52]. In a study on 34 LTx patients, elevated LLMI levels in BAL samples correlated significantly with abnormal 24 h-pH-m [27]. One study on 33 children with GER-related respiratory diseases found no increase in LLMI compared with controls [21]. A large study on 446 children with respiratory disorders thought to be GER-associated showed no associations between LLMI and various parameters in double channel 24 h-pH-m [53]. Therefore, LLMI in BAL seems to be of limited value in assessing GER-associated respiratory diseases, except perhaps in LTx patients.

Among 30 children with asthma-like symptoms, those with GER had higher levels of IL-8, myeloperoxidase, and elastase in BAL than those without GER [52]. Children with chronic lung diseases have been shown to have a positive correlation between IL-8 and protein carbonyl levels in BAL and proximal reflux events in 24 h-pH-m [48]. In LTx patients, IL-8 was found to be significantly elevated in those with elevated bile acids, but not IL-15 [31, 32]. Another study on 8 LTx patients which underwent antireflux surgery measured numerous inflammatory markers but found only that the level of IL-1-beta had decreased whereas the level of interferon-gamma had increased. However, these results were most likely confounded by the low number of participants and the high number of biomarkers studied [24]. 

Measurements of surfactant in BAL showed that dipalmitoylphosphatidylcholine did not differ between children with reflux esophagitis, cough, and healthy controls [20]. In another study, however, children with GER-associated chronic respiratory diseases were shown to have prominently reduced levels of surfactant-protein- (SP-) A and reduced levels of SP-D, compared with healthy controls [54]. Further studies are needed to evaluate the potential role of surfactant proteins as biomarkers to differentiate between chronic respiratory diseases with and without GER.

One study on bronchial aspirate in GER patients was found. Bronchial aspirate differs from BAL in that it does not introduce any foreign fluid into the lung but aspirates the pulmonary lining fluid directly. This study showed that GER patients have higher lactate dehydrogenase levels compared to healthy controls as well as a lower pH. Their lung function was also decreased compared to healthy controls [41]. 

To summarize, bile acids in bronchoalveolar lavage predict GER-induced transplant rejection in LTx patients. LLMI seems to be of limited value in assessing GER-associated respiratory diseases. GER can likely induce inflammation in the lungs and seems to have a different inflammatory profile than asthma.

## 6. Exhaled Breath Condensate Biomarkers

Exhaled breath condensate is a fluid biosample collected by guiding exhaled air into a condenser system, which cools the air and forms a condensate of the humidity in the air [55].

A recent study on pepsin levels in EBC samples from idiopathic pulmonary fibrosis patients did not show a significant elevation in pepsin, even though they had more GER symptoms on a questionnaire, compared to pulmonary fibrosis patients of a known cause [56]. The drawbacks of this study were, however, that it had few participants and used “home-made” equipment for EBC collection. Carpagnano et al. [7] showed elevated IL-4 in the EBC of asthmatics, irrespective of GER status. Conversely, IL-6 and 8-isoprostane were elevated in GER patients, irrespective of asthma status. These findings in EBC samples were similar to those in the sputum samples [7]. Another study found 8-isoprostane to be elevated in asthmatics, especially if they had comorbid GER, compared with healthy controls. This elevation was lowered significantly with proton pump inhibitor (PPI) treatment among the asthmatics with GER, but not among the asthmatics without GER [12].

Asthmatics with GER showed a lower pH in EBC than asthmatics without GER. PPI treatment seemed to elevate this low pH to a level similar to other non-GER asthmatics [10, 12]. In a 6-month prospective study on chronic obstructive pulmonary disease patients, a lower pH in EBC at baseline did not predict exacerbation frequency during followup. However, a lower pH in EBC was associated with GER status, and those with GER did have more exacerbations, suggesting this might be a false negative finding [57]. The EBC pH in chronic cough patients with GER was lower than in healthy controls [22]. 

Two studies from the same research group on calcium and magnesium in EBC showed conflicting results. The former study did not show a direct relationship between these electrolytes among 66 children with asthma, GER, or healthy children. The magnesium to calcium ratio, however, was lower in both children with asthma and those with GER. The later and larger study found calcium and magnesium to be elevated among children with GER, and inversely related to the EBC pH [9, 11]. Another study found levels of chloride to be lower in the EBC of 5 GER-induced chronic cough patients compared with 16 healthy controls [22]. As chloride and a higher pH have antitussive properties, the decrease in chloride and pH might contribute to the chronic cough in certain GER patients. How GER lowers chloride and even pH in the respiratory tract remains to be studied.

In summary, pulmonary inflammation in GER patients seems to be induced by different pathways than in asthma patients, as assessed by exhaled breath condensate. The pH value of EBC seems to be lowered in GER patients, and electrolyte disturbances have also been described.

## 7. Fractional Exhaled Nitric Oxide

Fractional exhaled nitric oxide (FeNO) has frequently been shown to be elevated in patients with classical asthma, and more recent evidence has accumulated that it is also a marker of eosinophilic inflammation in patients with chronic cough (eosinophilic bronchitis). Subjects with chronic cough and GER seem to have significantly lower FeNO than those with asthma without GER [14]. A cross-sectional study of 20 GER subjects with cough or asthma, however, did not support this conclusion, as it was found that asthma rather than GER caused an elevation in FeNO levels [13].

The presence of GER has been shown to improve the specificity of FeNO for diagnosing eosinophilic airway inflammation. Indeed, FeNO seems only to be of use among chronic cough patients in diagnosing eosinophilic airway inflammation when GER is present [23]. In asthmatic children with GER, FeNO levels were lower than in non-GER asthmatic children, suggesting that inhalation of gastric contents may interfere with FeNO production in the airways [15].

## 8. Particles in Exhaled Air

Particles in exhaled air (PEx) are formed when the respiratory lining fluid in the small airways erupts as the airways expand, for example, during inhalation after a deep exhalation [58]. This breathing maneuver is used when PEx are sampled, using an instrument designed especially for this purpose. The formed particles follow the exhaled air, the number of PEx is calculated, and the particles are sampled on a teflon filter by impaction [59]. 

No studies on particles in exhaled air (PEx) in GER were found. The main constituents of PEx are phospholipids originating from the surfactant. A previous study indicated increased protonated (H^+^) adduct formation of the major phospholipids among smokers, possibly related to alterations of the pH of the respiratory tract lining fluid (unpublished data). So far, there are no data on phospholipid alterations of the surfactant in GER in humans but gastric fluid aspiration is likely to influence the chemical composition and the pH of the respiratory tract lining fluid. Whether this also occurs in the distal airways, reflected by PEx, remains to be elucidated. 

## 9. Conclusions

Numerous studies evaluating biomarkers in GER-related respiratory conditions have been carried out. This paper focused mostly on induced sputum, BAL, and EBC samples. Our conclusion is that GER patients with respiratory diseases seem to have a different biochemical profile compared to similar patients without GER. Inflammatory markers differ in asthmatics based on GER status, tachykinins are elevated in GER-related cough patients, and bile acids are elevated in LTx patients with GER. However, the studies on each biomarker in a specific biosample are often small and few in number, making definite conclusions on the importance of these problematic markers.

The studies reviewed here were both on children and adults. Although these studies seem to be similar in many ways, some differences can be found. For example, pepsin seems to be more common in induced sputum in the pediatric population than the adult population [19, 43]. Children with neurodisability have a high incidence of reflux aspiration and comprise a specific group of GER patients [60]. Therefore, it is important not to draw conclusions about the adult population from studies on children, and vice versa.

Studies on the lipid laden macrophage index (LLMI) in BAL samples showed conflicting results. LLMI has been found to be elevated in pulmonary diseases with no evidence of aspiration, which also makes it nonspecific [37, 61]. The usefulness of LLMI in BAL for diagnosing GER seems therefore to be minimal.

The presence of pepsin in biosamples from the respiratory tract can perhaps not be considered as pathognomonic for a GER-related pulmonary aspiration. Diagnostic methods for pepsin are different and recently it was shown that pepsinogen produced in the lungs could be a confounding factor. As quite a few studies only measure the presence or absence of pepsin, further studies should rather assess the exact magnitude of pepsin in these samples.

The pathogenesis behind the associations of GER with respiratory diseases seems to be different between different respiratory diseases. This is reflected in the different biochemical findings. In chronic cough, pepsin and bile acids are usually not elevated, but tachykinins such as substance P and neurokinin A are, indicating that a vagally-mediated bronchoconstrictive reflex is responsible. In contrast, LTx patients with GER have significantly elevated levels of pepsin and bile acids, indicating gastric fluid aspiration as a predominant causative factor. This difference in pathogenesis has to be thought of when planning studies on biomarkers in GER-associated respiratory diseases.

Reviewing respiratory biomarkers in GER leads to several perplexities. First and foremost is the wide definition of GER, which is basically the presence of bothersome symptoms caused by reflux of gastric contents [2]. GER is diagnosed based on widely different questionnaires, sometimes stressing the importance of sleep-related GER and sometimes not. Doing 24-hour esophageal pH monitoring (24 h-pH-m) is sometimes based on only one level of monitoring 5 cm above the lower esophageal sphincter, but sometimes higher (15 cm) as well [62]. It has also been pointed out that one negative 24 h-pH-m is not enough to eliminate the possibility of GER. As many as three nights might be needed. Also, in the case of EBC, these measurements have shown to have little reproducibility and are poorly standardized, making their usefulness currently limited. For the application of EBC to become more successful, collection methods and biomarker analyses in EBC samples need to become more standardized. This standardization would in turn make research collaborations easier, which is crucial for further development of this method [55, 63].

As the symptoms of GER-induced respiratory disorders often mimic other common respiratory disorders, a reliable test to identify GER-induced respiratory disorders needs to be developed. Such a test should ideally be noninvasive, with a high positive predictive value, low intraindividual variability, and change with effective treatment. In this context, the measurement of a biomarker or a set of biomarkers from the respiratory tract is of special interest. The BAL samples, which have been studied the most, are too invasive to be of use in populations other than LTx patients. EBC samples are promising, but need further evaluation and standardization [55]. The newly developed PEx measurements remain to be studied further.

## Figures and Tables

**Table 1:** Biomarkers studied in gastroesophageal reflux with respiratory illnesses.

Biosample	Serum	Sputum		Bronchoalveolar lavage		Exhaled air	Exhaled breath condensate
Studies found	22	34		71		7	16
Studies reviewed	2	11		21		4	8
Biomarkers studied	Eosinophil cationic protein	Bile acids	Pepsin	Bile acids	Pepsin	FeNO	Pepsin
	Neurotrophin 3	LLMI	IL-4, IL-6	LLMI	IL-1, IL-8, IL-15		IL-4, IL-6
	BDNF	Substance P	Neurokinin A	IFN-gamma	Myeloperoxidase		8-Isoprostane
	Nerve growth factor	Nerve growth factor	Mast cell tryptase	Lactate dehydrogenase	Protein carbonyls		pH
		8-Isoprostane	Histamine	DPPC	SP-A, SP-D		Calcium
		Prostaglandin D2	Fibrinogen	Elastase	pH		Magnesium
		Eosinophil cationic protein					Chloride

## Abbreviations

24 h-pH-m: 24 hour esophageal pH monitoring
BAL: Bronchoalveolar lavage
EBC: Exhaled breath condensate
ECP: Eosinophil cationic protein
GER: Gastroesophageal reflux
IL: Interleukin
LLMI: Lipid laden macrophage index
LTx: Lung transplant
MII-pH: Multichannel intraluminal impedance pH monitoring
NGF: Nerve growth factor
PEx: Particles in exhaled air.

## References

[1] Vakil N (2010). Disease definition, clinical manifestations, epidemiology and natural history of GERD. *Best Practice and Research*.

[2] Vakil N, Van Zanten SV, Kahrilas P (2006). The Montreal definition and classification of gastroesophageal reflux disease: a global evidence-based consensus. *American Journal of Gastroenterology*.

[3] Emilsson OI, Bengtsson A, Franklin KA (2012). Nocturnal gastroesophageal reflux, asthma and symptoms of obstructive sleep apnoea: a longitudinal, general population study. *European Respiratory Journal*.

[4] Perng DW, Chang KT, Su KC (2007). Exposure of airway epithelium to bile acids associated with gastroesophageal reflux symptoms: a relation to transforming growth factor-*β*1 production and fibroblast proliferation. *Chest*.

[5] Patterson RN, Johnston BT, Ardill JES, Heaney LG, McGarvey LPA (2007). Increased tachykinin levels in induced sputum from asthmatic and cough patients with acid reflux. *Thorax*.

[6] Di Lorenzo G, Mansueto P, Esposito-Pellitteri M (2007). The characteristics of different diagnostic tests in adult mild asthmatic patients: comparison with patients with asthma-like symptoms by gastro-oesophageal reflux. *Respiratory Medicine*.

[7] Carpagnano GE, Resta O, Ventura MT (2006). Airway inflammation in subjects with gastro-oesophageal reflux and gastro-oesophageal reflux-related asthma. *Journal of Internal Medicine*.

[8] Borrelli O, Battaglia M, Galos F (2010). Non-acid gastro-oesophageal reflux in children with suspected pulmonary aspiration. *Digestive and Liver Disease*.

[9] Banović S, Navratil M, Vlasic Ž, Topić RZ, Dodig S (2011). Calcium and magnesium in exhaled breath condensate of children with endogenous and exogenous airway acidification. *Journal of Asthma*.

[10] Liu L, Teague WG, Erzurum S (2011). Determinants of exhaled breath condensate pH in a large population with asthma. *Chest*.

[11] Dodig S, Vlasic Z, Čepelak I, Topić RZ, Turkalj M, Nogalo B (2009). Magnesium and calcium in exhaled breath condensate of children with asthma and gastroesophageal reflux disease. *Journal of Clinical Laboratory Analysis*.

[12] Shimizu Y, Dobashi K, Zhao JJ (2007). Proton pump inhibitor improves breath marker in moderate asthma with gastroesophageal reflux disease. *Respiration*.

[13] Parameswaran K, Allen CJ, Kamada D, Efthimiadis A, Anvari M, Hargreave FE (2001). Sputum cell counts and exhaled nitric oxide in patients with gastroesophageal reflux, and cough or asthma. *Canadian Respiratory Journal*.

[14] Kowal K, Bodzenta-Lukaszyk A, Zukowski S (2009). Exhaled nitric oxide in evaluation of young adults with chronic cough. *Journal of Asthma*.

[15] Silvestri M, Mattioli G, Defi Lippi AC (2004). Correlations between exhaled nitric oxide levels and pH-metry data in asthmatics with gastro-oesophageal reflux. *Respiration*.

[16] Qiu Z, Yu L, Xu S (2011). Cough reflex sensitivity and airway inflammation in patients with chronic cough due to non-acid gastro-oesophageal reflux. *Respirology*.

[17] Chaudhuri R, McMahon AD, McSharry CP (2005). Serum and sputum neurotrophin levels in chronic persistent cough. *Clinical and Experimental Allergy*.

[18] Grabowski M, Kasran A, Seys S (2011). Pepsin and bile acids in induced sputum of chronic cough patients. *Respiratory Medicine*.

[19] Decalmer S, Stovold R, Houghton LA (2012). Chronic cough: relationship between microaspiration, gastroesophageal reflux, and cough frequency. *Chest*.

[20] Chang AB, Hills YC, Cox NC (2006). ‘Free’ surfactant in gastric aspirates and bronchoalveolar lavage in children with and without reflux oesophagitis. *Internal Medicine Journal*.

[21] Farrell S, McMaster C, Gibson D, Shields MD, McCallion WA (2006). Pepsin in bronchoalveolar lavage fluid: a specific and sensitive method of diagnosing gastro-oesophageal reflux-related pulmonary aspiration. *Journal of Pediatric Surgery*.

[22] Niimi A, Nguyen LT, Usmani O, Mann B, Chung KF (2004). Reduced pH and chloride levels in exhaled breath condensate of patients with chronic cough. *Thorax*.

[23] Pacheco A, Faro V, Cobeta I, Royuela A, Molyneux I, Morice AH (2011). Gastro-oesophageal reflux, eosinophilic airway inflammation and chronic cough. *Respirology*.

[24] Fisichella PM, Davis CS, Lowery E (2012). Pulmonary immune changes early after laparoscopic antireflux surgery in lung transplant patients with gastroesophageal reflux disease. *Journal of Surgical Research*.

[25] Fisichella PM, Davis CS, Lundberg PW (2011). The protective role of laparoscopic antireflux surgery against aspiration of pepsin after lung transplantation. *Surgery*.

[26] Mertens V, Blondeau K, Van Oudenhove L (2011). Bile acids aspiration reduces survival in lung transplant recipients with BOS despite azithromycin. *American Journal of Transplantation*.

[27] Hopkins PM, Kermeen F, Duhig E (2010). Oil red O stain of alveolar macrophages is an effective screening test for gastroesophageal reflux disease in lung transplant recipients. *Journal of Heart and Lung Transplantation*.

[28] Mertens V, Blondeau K, Pauwels A (2009). Azithromycin reduces gastroesophageal reflux and aspiration in lung transplant recipients. *Digestive Diseases and Sciences*.

[29] Blondeau K, Mertens V, Vanaudenaerde BA (2008). Gastro-oesophageal reflux and gastric aspiration in lung transplant patients with or without chronic rejection. *European Respiratory Journal*.

[30] Blondeau K, Mertens V, Vanaudenaerde BA (2009). Nocturnal weakly acidic reflux promotes aspiration of bile acids in lung transplant recipients. *Journal of Heart and Lung Transplantation*.

[31] Vos R, Blondeau K, Vanaudenaerde BM (2008). Airway colonization and gastric aspiration after lung transplantation: do birds of a feather flock together?. *Journal of Heart and Lung Transplantation*.

[32] D'Ovidio F, Mura M, Tsang M (2005). Bile acid aspiration and the development of bronchiolitis obliterans after lung transplantation. *Journal of Thoracic and Cardiovascular Surgery*.

[33] Timms CJ, Yates DH, Thomas PS (2011). Diagnosing GORD in respiratory medicine. *Frontiers in Pharmacology*.

[34] De Giorgi F, Palmiero M, Esposito I, Mosca F, Cuomo R (2006). Pathophysiology of gastro-oesophageal reflux disease. *Acta Otorhinolaryngologica Italica*.

[35] Peitz U, Wex T, Vieth M (2011). Correlation of serum pepsinogens and gastrin-17 with atrophic gastritis in gastroesophageal reflux patients: a matched-pairs study. *Journal of Gastroenterology and Hepatology*.

[36] Jovov B, Que J, Tobey NA, Djukic Z, Hogan BLM, Orlando RC (2011). Role of e-cadherin in the pathogenesis of gastroesophageal reflux disease. *American Journal of Gastroenterology*.

[37] Corwin RW, Irwin RS (1985). The lipid-laden alveolar macrophage as a marker of aspiration in parenchymal lung disease. *American Review of Respiratory Disease*.

[38] Nussbaum E, Maggi JC, Mathis R, Galant SP (1987). Association of lipid-laden alveolar macrophages and gastroesophageal reflux in children. *Journal of Pediatrics*.

[39] Ahrens P, Noll C, Kitz R, Willigens P, Zielen S, Hofmann D (1999). Lipid-laden alveolar macrophages (LLAM): a useful marker of silent aspiration in children. *Pediatric Pulmonology*.

[40] Fisichella PM, Davis CS, Kovacs EJ (2012). A review of the role of GERD-induced aspiration after lung transplantation. *Surgical Endoscopy and Other Interventional Techniques*.

[41] Mise K, Capkun V, Jurcev-Savicevic A, Sundov Z, Bradaric A, Mladinov S (2010). The influence of gastroesophageal reflux in the lung: a case-control study. *Respirology*.

[42] Pauwels A, Decraene A, Blondeau K (2012). Bile acids in sputum and increased airway inflammation in patients with cystic fibrosis. *Chest*.

[43] Ervine E, McMaster C, McCallion W, Shields MD (2009). Pepsin measured in induced sputum—a test for pulmonary aspiration in children?. *Journal of Pediatric Surgery*.

[44] Elabiad MT, Zhang J (2011). Detection of pepsinogen in the neonatal lung and stomach by immunohistochemistry. *Journal of Pediatric Gastroenterology and Nutrition*.

[45] Gerson KD, Foster CD, Zhang P, Zhang Z, Rosenblatt MM, Guttentag SH (2008). Pepsinogen C proteolytic processing of surfactant protein B. *The Journal of Biological Chemistry*.

[46] Köksal D, Özkan B, Pimpek C, Köksal AP, Adaçkýran Y, Papmaz N (2005). Lipid-laden alveolar macrophage index in sputum is not useful in the differential diagnosis of pulmonary symptoms secondary to gastroesophageal reflux. *Archives of Medical Research*.

[47] Honeybourne D, Babb J, Bowie P (2001). British Thoracic Society guidelines on diagnostic flexible bronchoscopy. *Thorax*.

[48] Starosta V, Kitz R, Hartl D, Marcos V, Reinhardt D, Griese M (2007). Bronchoalveolar pepsin, bile acids, oxidation, and inflammation in children with gastroesophageal reflux disease. *Chest*.

[49] Church AC, Goldsmith K, Sivasothy P (2010). Aspiration and development of subglottic stenosis in patients with Wegeners granulomatosis. *Journal of Laryngology and Otology*.

[50] Sacco O, Silvestri M, Sabatini F, Mattioli G, Rossi GA (2000). Bronchoalveolar lavage and esophageal pH monitoring data in children with “difficult to treat” respiratory symptoms. *Pediatric Pulmonology*.

[51] Bibi H, Khvolis E, Shoseyov D (2001). The prevalence of gastroesophageal reflux in children with tracheomalacia and laryngomalacia. *Chest*.

[52] Sacco O, Silvestri M, Sabatini F (2006). IL-8 and airway neutrophilia in children with gastroesophageal reflux and asthma-like symptoms. *Respiratory Medicine*.

[53] Kitz R, Boehles HJ, Rosewich M (2012). Lipid-laden alveolar macrophages and pH monitoring in gastroesophageal reflux-related respiratory symptoms. *Pulmonary Medicine*.

[54] Griese M, Maderlechner N, Ahrens P, Kitz R (2002). Surfactant proteins A and D in children with pulmonary disease due to gastroesophageal reflux. *American Journal of Respiratory and Critical Care Medicine*.

[55] Rosias P (2012). Methodological aspects of exhaled breath condensate collection and analysis. *Journal of Breath Research*.

[56] Fahim A, Dettmar PW, Morice AH, Hart SP (2011). Gastroesophageal reflux and idiopathic pulmonary fibrosis: a prospective study. *Medicina*.

[57] Terada K, Muro S, Sato S (2008). Impact of gastro-oesophageal reflux disease symptoms on COPD exacerbation. *Thorax*.

[58] Almstrand AC, Ljungström E, Lausmaa J, Bake B, Sjövall P, Olin AC (2009). Airway monitoring by collection and mass spectrometric analysis of exhaled particles. *Analytical Chemistry*.

[59] Almstrand AC, Bake B, Ljungström E (2010). Effect of airway opening on production of exhaled particles. *Journal of Applied Physiology*.

[60] Trinick R, Johnston N, Dalzell AM, McNamara PS (2012). Reflux aspiration in children with neurodisability—a significant problem, but can we measure it?. *Journal of Pediatric Surgery*.

[61] Knauer-Fischer S, Ratjen F (1999). Lipid-laden macrophages in bronchoalveolar lavage fluid as a marker for pulmonary aspiration. *Pediatric Pulmonology*.

[62] Fahim A, Crooks M, Hart SP (2011). Gastroesophageal reflux and idiopathic pulmonary fibrosis: a review. *Pulmonary Medicine*.

[63] Davis CS, Gagermeier J, Dilling D (2010). A review of the potential applications and controversies of non-invasive testing for biomarkers of aspiration in the lung transplant population. *Clinical Transplantation*.

